# Pruritus associated with systemic sclerosis: a systematic review of treatment-based clinical trials

**DOI:** 10.1007/s00296-026-06190-5

**Published:** 2026-06-22

**Authors:** Kiana Mortezaei, Rishika Pasula, Meryem Nursoy, Amit Syal, Ioannis Panayiotou, Geeta Kumari, Andreas Chatzitoffis, Pavle Milutinovic, Olajumoke O. Fadugba, Chris T. Derk

**Affiliations:** 1 Drexel College of Medicine, Philadelphia, PA USA; 2 Hull University Teaching Hospitals NHS Trust, Hull, UK; 3 Sidney Kimmel Medical College at Thomas Jefferson University, Philadelphia, PA USA; 4 School of Medicine, University of Cyprus, Nicosia, Cyprus; 5 Department of Medicine, University of Pennsylvania, Philadelphia, PA USA; 6 Department of Psychiatry, University of Cyprus, Nicosia, Cyprus; 7 Division of Pulmonary, Allergy and Critical Care, University of Pennsylvania, Philadelphia, PA USA; 8 Division of Rheumatology, University of Pennsylvania, Philadelphia, PA USA

**Keywords:** Systemic sclerosis, Scleroderma, Pruritus, Itch, Rituximab, Quality of life, Naltrexone, Diffuse cutaneous scleroderma, Limited cutaneous scleroderma, Outcome assessment

## Abstract

This paper aims to examine the latest research on treatments for itch (pruritus) in people with systemic sclerosis (SSc), identify areas where information is lacking, and propose suggestions for future studies.A comprehensive literature search was performed in PubMed and Scopus in accordance with the Preferred Reporting Items for Systematic Reviews and Meta-Analyses (PRISMA) guidelines to identify studies evaluating therapeutic interventions for pruritus in SSc. Eligible studies included randomized controlled trials, cohort studies, and case series comprising a minimum of three patients. Data extraction encompassed study design, patient demographics, pruritus assessment instruments including validated and non-validated measures, and clinical outcomes. Studies were excluded if they concerned localized scleroderma, irrelevant populations, case reports, systematic reviews, lacking relevant outcome data, or incomplete trials. Ten studies met criteria, evaluating immunomodulatory agents, mast cell stabilizers, opioid receptor antagonist, and lysophosphatidic acid receptor antagonists. Pruritus assessment varied and was often secondary to fibrosis outcomes. Oral lenabasum improved pruritus in a phase II trial; low-dose opioid receptor modulators and rituximab also showed qualitative improvement. Pruritus is a frequently overlooked yet clinically significant symptom in systemic sclerosis (SSc) that negatively impacts patients’ quality of life. Existing evidence regarding effective therapeutic options remains limited. Future research should prioritize pruritus as a predefined outcome and utilize validated assessment instruments to inform treatment strategies and enhance patient quality of life.

## Introduction

Pruritus, commonly referred to as itching, is an unpleasant sensory experience that provokes the urge to scratch. It is a common and distressing symptom not only in dermatologic diseases but also in autoimmune disorders such as systemic lupus erythematosus (SLE) and systemic sclerosis (SSc) [1]. The sensation of pruritus can range from mild to debilitating and is often associated with significant impairment in quality of life, including sleep disturbance and emotional distress [2]. The psychological burden of chronic pruritus is substantial and characterized by bidirectional relationships with psychiatric disorders [3]. Pruritus has been associated with multiple psychiatric disorders, including depression, anxiety, somatoform disorders, obsessive-compulsive disorder, and psychosis [4]. Even when pruritus arises from autoimmune disease, symptoms may be exacerbated by psychosocial stressors and emotional triggers. Compared with individuals without itch, patients with chronic pruritus report higher levels of stress, anxiety, depression, and perceived stigmatization [2]. The vicious itch-scratch cycle may further exacerbate underlying disease manifestations and psychiatric symptoms, including depression, anxiety, and suicidal ideation [5]. In dermatological conditions, pruritus is frequently associated with inflammation, dryness, and disruption of the skin barrier [6]. However, in autoimmune diseases such as SLE and SSc, substantial itching may occur even in the absence of visible skin lesions, reflecting underlying immune dysregulation and chronic inflammation [7]. The mechanisms driving pruritus in these disorders are complex and may involve abnormal activation of immune cells, mast cells, release of pro-inflammatory cytokines, and alterations in nerve signaling pathways [8]. Effective management of pruritus in autoimmune diseases requires a comprehensive understanding of its multifactorial origins, as well as individualized approaches that address both the physical and psychosocial aspects of the symptom [1]. From a mechanistic perspective, pruritus arises through both histamine-dependent and histamine-independent pathways. The histamine-dependent pathway involves activation of histamine-1 receptors and downstream phospholipase C beta 3 (PLCβ3), resulting in pruritic signaling and scratching behavior [6]. The histamine-independent pathway involves C fibers and protease-activated receptors (PARs) [6]. Pruritus is increasingly recognized as a significant but underappreciated symptom in SSc, a rare autoimmune connective tissue disease marked by excess collagen in the skin and other organs. Approximately 45% of patients with SSc report pruritus, with affected individuals demonstrating more severe skin, respiratory, and gastrointestinal involvement [8].

Although the exact pathogenesis of pruritus in SSc remains unclear, it has been reported that histamine, lysophosphatidic acid, and prostaglandin E2 are involved [9]. Histamine is a well-known mediator of itching, primarily acting through histamine-1 receptors to trigger the sensation of pruritus and promote scratching behavior [6]. In SSc, increased levels of histamine may result from immune cell activation and mast cell degranulation, contributing to the itching experience [8]. Lysophosphatidic acid (LPA) is another bioactive lipid that has been implicated in the development of pruritus; it can sensitize nerve endings and promote inflammation, thereby amplifying itch signals [8]. Prostaglandin E2 (PGE2), a product of arachidonic acid metabolism, plays a role in inflammation, potentially contributing to pruritus in affected individuals [8]. These mediators interact within complex immune and neural pathways, reflecting the multifactorial nature of pruritus in systemic sclerosis. Interleukin-31 has also emerged as a key cytokine mediator of pruritus, with preclinical models demonstrating its role in promoting fibrosis and T-helper-2 polarization in SSc [10]. Continued research is needed to fully elucidate these mechanisms and guide the development of targeted therapies for itch management in SSc patients. Pruritus is common and impactful in SSc, yet research and treatment options are limited. Treatments used for other pruritic conditions may not be effective for SSc. This systematic review summarizes current studies on pruritus treatment in SSc, highlights gaps in the literature, and suggests directions for future research.

## Methods

### Search strategy

**Diagram 1:**
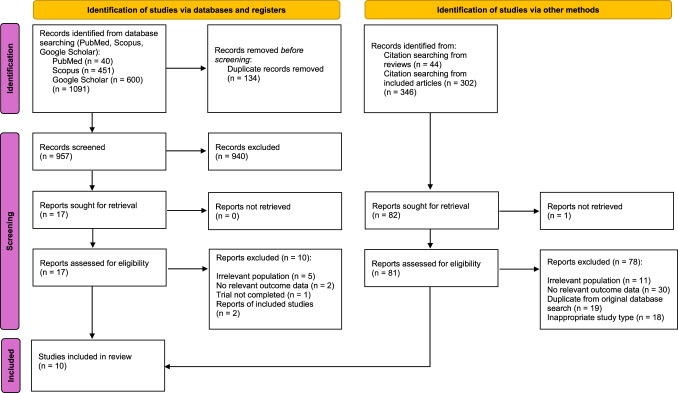
SSc related Pruritus PRISMA flow diagram detailing search

This systematic review was conducted in accordance with the Preferred Reporting Items for Systematic Reviews and Meta-Analyses (PRISMA) guidelines [11]. A literature search was performed using PubMed and Scopus databases with the search terms systemic sclerosis OR scleroderma, pruritus OR itch, and treatment OR therapy OR management OR intervention. Additional study design search terms applied in the PubMed database included case reports, case series, open-label studies, and randomized controlled trials. The Scopus search was limited to articles and reviews. All database searches were restricted to human studies. The search was undertaken in October 2025 independently by five investigators (K.M., R.P., M.N, A.S. and G.K.), with final verification by C.T.D. and G.K. Duplicate records were identified and removed using EndNote (EndNote 2025). The initial screening was performed by title and abstract to identify relevant studies, followed by a full-text review to determine eligibility based on inclusion and exclusion criteria. Relevant systematic and narrative reviews were also examined to identify additional original studies through reference review. The reference lists of included articles were further reviewed to identify additional eligible studies. Final consensus was reached following full-text review (Diagram 1).

### Study selection

We included original articles in English that documented treatment responses for pruritus in individuals with SSc. Randomized controlled trials, cohort studies, and case series with at least three patients were eligible for inclusion. Inclusion criteria were original research studies involving patients with SSc who were assessed for pruritus before and after treatment using either validated or non-validated measures. Exclusion criteria were abstracts, case reports, systematic or narrative reviews, duplicate studies, and discontinued trials.

### Data extraction and synthesis

**Fig. 1:**
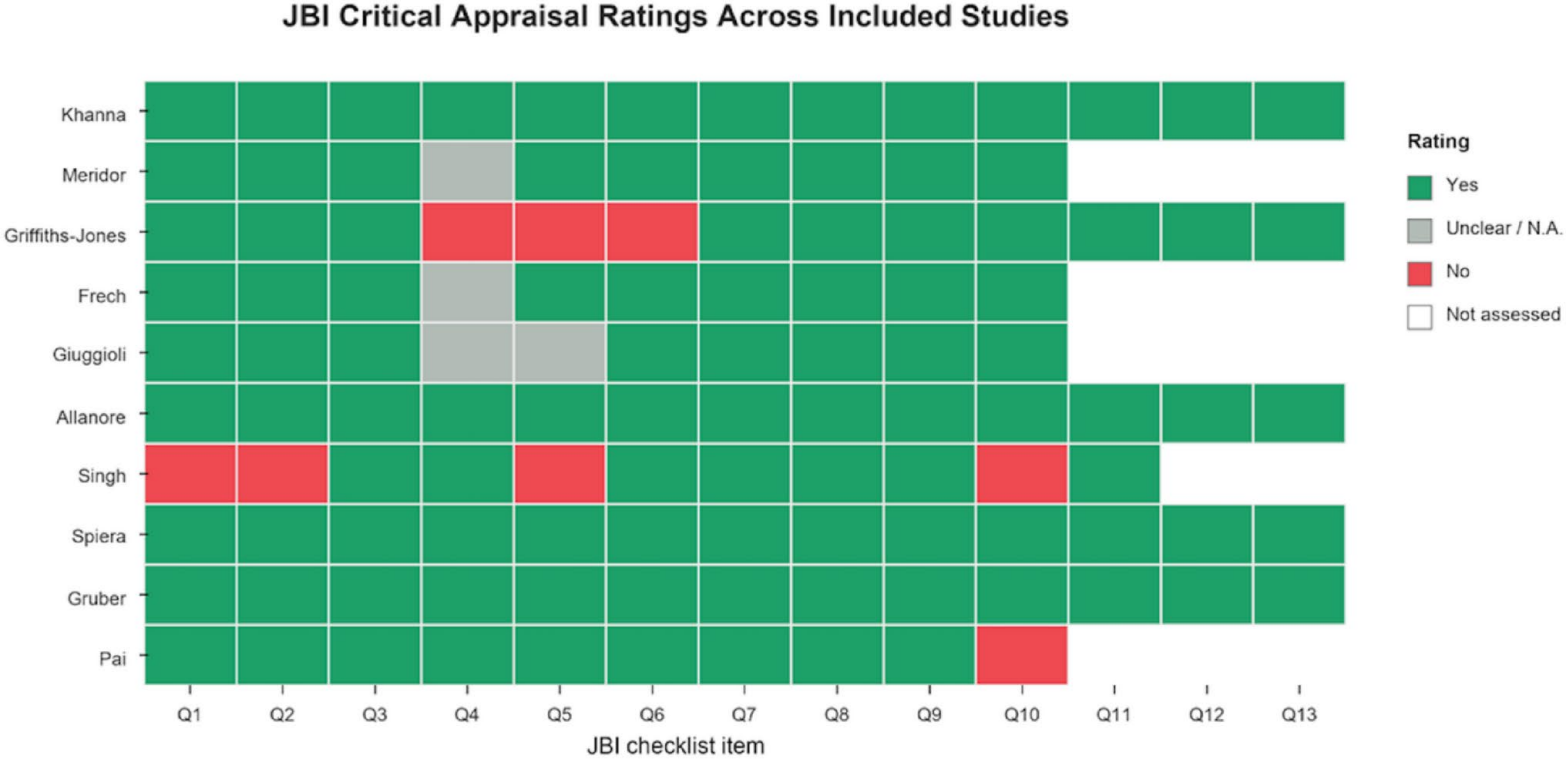
JBI checklist ratings across included studies evaluating systemic sclerosis–associated pruritus. Each row represents one study, and each column corresponds to a Joanna Briggs Institute (JBI) checklist question. Cells are color-coded by response category: green indicates “Yes,” gray indicates “Unclear / Not Applicable,” and red indicates “No.” This visualization highlights variation in methodological quality across studies, with generally high adherence to JBI criteria but inconsistent reporting in select domains

Data extraction included lead author, country, year of publication, study design, study aim, patient demographics, pruritus assessment instrument including validated and non-validated measures, study outcomes, and statistical significance. The Joanna Briggs Institute (JBI) appraisal tool was used to assess the risk of bias. The JBI tool evaluates the methodological quality of studies using a structured checklist that addresses key domains such as population, exposure measurement, confounding factors, and outcome measurement. For all included articles, each item was scored as Yes [1] or No/Not applicable/Unclear (0) by two independent appraisers. The number of appraisal items varied according to study type: cohort studies (11 items) [12], case series (10 items) [13], and case-control (9 items). The scores for each study are presented in Table 1. Methodological quality assessment results are summarized in Fig. 1.

## Results

We screened a total of 491 records from PubMed and Scopus, with three additional studies identified through manual review of references, for trials in which pruritus management was an outcome measure of systemic sclerosis (SSc). A total of ten trials and case series were identified (Diagram 1) and summarized in a table format (Table 1). Methodological quality was summarized in Fig. 1.

In 2020, a 12-week double-blind, placebo-controlled Phase II trial conducted by *Spiera* et al. assessed the safety and efficacy of Lenabasum, an oral cannabinoid, in 42 patients with diffuse SSc (disease duration ≤ 6 years, ages 18–69) on stable standard care. Efficacy was evaluated with the Composite Response Index in Systemic Sclerosis (CRISS) score, incorporating mRSS, Health Assessment Questionnaire- Disability Index (HAQDI), global assessments, and lung function [14]. Treatment with lenabasum led to greater improvements in CRISS scores and mRSS, and reduced pruritus severity per the 5-D itch scale from week 4 onward. For patients treated with lenabasum, the 5-D itch score improved by − 1.8 ± 1.0 at week 12 (baseline 10.7 ± 4.4 vs. 12.9 ± 5.3 in placebo), reaching significance by one-sided mixed model repeated measures analysis (*P* ≤ 0.05), but not two-sided analysis (*P* ≤ 0.10) [14]. Lenabasum improved severity of pruritus which may be related to its reported effects on IL-31, however limitations of this study include a small sample size and short duration of treatment.

In 2015, a prospective, open-label, uncontrolled case series by *Giuggioli* et al. studied the effect of rituximab (RTX), an anti-CD20 monoclonal antibody, in the treatment of patients with SSc. This study had a sample size of 10 patients, half with lc-SSc and the other half with dc-SSc. The cohort was 60% female with a mean age of 46 years. Patients received rituximab at 375 mg/m^2^ for four infusions per cycle, with follow-up six months post treatment. The efficacy of RTX was evaluated using the mRSS, pulmonary function testing, and pain VAS [15]. The results suggested the B cell depletion significantly improved the severity of skin sclerosis in patients, and secondarily, clinically improved hypermelanosis, calcinosis, and pruritus as well. Pruritus improved in most patients treated with rituximab (6 of 8), although outcomes were reported qualitatively rather than with numeric itch scores [15]. Rituximab showed benefit in pruritus in most treated patients, however the qualitative nature of pruritus reporting and the absence of a validated instrument to measure pruritus limits the strength of the findings.

In a 1995 case series from Pai et al., five female patients with progressive SSc were treated with dexamethasone pulse therapy. The patients evaluated were between the ages of 30 and 60 years and had a history of progressive SSc [16]. Each patient received 100 mg of dexamethasone in 500 mL of 5% dextrose IV over three hours, administered three consecutive days once every month. The efficacy of dexamethasone was measured by evaluating improvement in Raynaud’s phenomenon, itching, dysphagia, changes in the induration of skin, erythrocyte sedimentation rate, and evidence of dermal fibrosis in skin biopsies. All patients experienced symptomatic and clinical improvement following dexamethasone therapy, with 2 patients reporting reduction in itching [16]. Two of five patients in the study reported a reduction in itching suggesting a potential role for dexamethasone pulse therapy in managing SSc associated pruritus. However, the small sample size and lack of validated instrument to quantify pruritus makes it difficult to draw firm conclusions regarding its efficacy.

In 2018, a double-blind, randomized, placebo-controlled trial by *Allanore* et al. evaluated the effectiveness of the lysophosphatidic acid (LPA) receptor antagonist SAR100842 in patients with dc-SSc. The study included 32 participants with SSc diagnosed within the previous 36 months; the average age was around 49 years, and approximately 60% were female. Researchers measured clinical efficacy using the mRSS [17]. Although SAR100842 was well tolerated and resulted in lower mRSS compared to the placebo, the difference was not statistically significant. The study also assessed pruritus severity through patient ratings on a 0–10 cm VAS, and despite low baseline severity, patients receiving the LPA receptor antagonist SAR100842 reported less severe pruritus than those in the placebo group (mean change − 0.37 ± 3.92 vs. + 0.25 ± 1.79). Furthermore, there was reduced pruritus severity for continuously treated patients (SAR100842/ SAR100842) compared to placebo/ SAR100842 patients (mean ± SD change − 1.38 ± 2.85 versus − 0.84 ± 1.67) [17]. LPA receptor antagonist SAR100842 showed reduced pruritus severity, however the results did not report statistical testing for pruritus, limiting conclusions to the efficacy for pruritus.

A case series from 2011 by *Frech* et al. evaluated three female patients with SSc and chronic pruritus of at least 6 weeks to understand the efficacy of low-dose naltrexone. Two patients had diffuse cutaneous scleroderma, and one patient had limited cutaneous scleroderma. These patients were previously unresponsive to treatment with antihistamines. Low-dose naltrexone was initiated at 2 mg nightly. It was titrated to a maximum of 4.5 mg over the trial duration of two months to assess the difference in pruritus, with two patients reaching the target dose and one remaining at 2 mg. The study demonstrated clinical improvement in pruritus, measured by the 10-point FACES scale, with two of three patients reporting complete resolution of itch. There was also improvement in the modified Rodman skin score (mRSS) [18]. These findings suggest that low-dose naltrexone may be a potential treatment for pruritus in SSc, however the small sample size highlights the need for larger controlled trials.

A Phase II double-blind, placebo-controlled randomized trial by *Khanna* et al. evaluated subcutaneous tocilizumab for safety and efficacy in SSc. Tocilizumab is an interleukin-6 targeting monoclonal antibody that may lower CCL18, a chemokine linked to SSc-related lung fibrosis. Eighty-seven patients with diffuse cutaneous systemic sclerosis (77% female, average age 48–51) were assigned to either tocilizumab or placebo for 48 weeks [19]. Efficacy was measured mainly by the mRSS, along with pruritus 5-D itch score, VAS, and Functional Assessment of Chronic Illness Therapy (FACIT). Tocilizumab resulted in improvement in skin scores, although differences did not reach statistical significance, and there was no significant difference in pruritus between groups [19]. The absence of improvement in itching with treatment of tocilizumab may suggest that itch in SSc may be independent of IL-6 mediated inflammation.

A case series by *Meridor* et al. involving four female patients diagnosed with SSc and experiencing severe pruritus evaluated the efficacy of low-dose oral naloxone (4.5 mg) for pruritus management. The participants, aged between 31 and 74, had either diffuse or limited cutaneous SSc. Pruritus was measured using the 5D itch scale, with a baseline mean score of 22.75 prior to treatment initiation. Following six and twelve months of therapy, the average pruritus score decreased to 7.5, yielding an overall mean reduction of 15.25 points in pruritus severity with a p score of 0.004 [9]. These findings suggest that low-dose oral naloxone may be a viable option for pruritus treatment in SSc, however a larger controlled trial is needed to confirm these results.

A 2023 retrospective study by *Singh* evaluated rituximab in 13 SSc patients (mostly female, median age 36, median disease duration 10 years). After four 500 mg infusions between January 2019 and June 2020, all showed significant improvement in cutaneous symptoms, with mRSS decreasing by 20% at 6 months and 37% at 12 months. Pruritus improved in all affected, although itch severity was not quantitatively assessed [20]. Although pruritus improved in all affected patients, the small sample size and lack of qualitative instrument to assess pruritus limits the strength of the findings.

In 2023, a United Kingdom multicenter Phase II trial by *Griffiths-Jones* et al. evaluated oral prednisone for early diffuse cutaneous systemic sclerosis in 35 adults. Originally designed as a double-blind trial, it became open-label due to COVID-19-related disruptions and was shortened. At 3 months, differences in HAQ-DI scores, mRSS, and 5-D itch scores favored prednisolone but were not statistically significant, with results underpowered due to the early termination [21]. Prednisolone was associated with lower itch scores however these findings are not statistically significant, limiting the strength of these conclusions.

A prospective, randomized, double-blind, placebo-controlled trial by Gruber et al. in 1991 evaluated the efficacy of ketotifen in SSc. Ketotifen is a mast-cell stabilizing agent and histamine H1 receptor antagonist evaluated for its effect on pruritus in a cohort of 24 patients with diffuse cutaneous SSc. As a drug commonly used in allergic disorders, ketotifen inhibits chemokine release by preventing mast cell degranulation [22]. The cohort consisted of 16 women and 8 men. The mean age was 46 years in the placebo group and 47 years in the ketotifen group. Pruritus was evaluated using a 0–3 scale over 5 months. At baseline, mean pruritus scores were 2.8 ± 1.1 in the ketotifen group and 1.7 ± 1.7 in the placebo group. After 24 weeks, scores decreased to 1.5 ± 1.5 and 1.3 ± 1.8, respectively; however, this reduction did not reach statistical significance. One significant finding was improvement in total skin score, measured by wheal and flare reactions, in the placebo group compared with the treatment group at week 12. The study indicated that ketotifen did not effectively prevent mast cell degranulation or reduce skin test reactivity, suggesting its lack of efficacy in treatment of pruritus [23].

## Discussion

Pruritus is a common and burdensome symptom in systemic sclerosis (SSc), yet it remains underrepresented as a prespecified endpoint in therapeutic studies. In this systematic review of treatment-based trials and case series, 159 patients with diffuse cutaneous systemic sclerosis and 9 patients with limited cutaneous systemic sclerosis were studied, making it difficult to draw meaningful comparative conclusions from the evidence. Furthermore, only a limited number of interventions evaluated pruritus outcomes in SSc, and assessment methods were highly heterogenous across studies. Most investigations prioritized skin fibrosis and internal organ manifestations, with pruritus frequently reported only as a secondary or exploratory endpoint and often without validated instruments, limiting comparability across studies and precluding meta-analysis. Given the substantial impact of pruritus on quality of life, sleep, and psychosocial well-being, greater attention to itch-specific outcomes in SSc research is warranted.

Interpretation of pruritus outcomes across studies is further complicated by substantial heterogeneity in assessment tools and reporting practices. Pruritus was evaluated using a range of instruments, including visual analog scales, the 5-D itch scale, and qualitative or descriptive measures, often without standardized baseline severity or uniform follow-up intervals. In several studies, pruritus was reported only as a secondary or exploratory outcome, limiting statistical power and interpretability. This variability underscores the need for consistent use of validated pruritus-specific instruments in future SSc trials.

Although not life-threatening, pruritus carries considerable psychosocial burden, as patients experiencing itch report higher rates of stress, anxiety, and depressive symptoms. The pathogenesis of pruritus in SSc is likely multifactorial, involving immune dysregulation and psychogenic influences. Within the reviewed studies, several therapies demonstrated potential benefit [2, 5].

From a mechanistic standpoint, several of the therapies evaluated in this review may influence pruritus through modulation of immune, inflammatory, or neuroimmune pathways. Agents such as lenabasum and rituximab exert immunomodulatory effects that may reduce inflammatory mediators implicated in itch signaling, while opioid receptor antagonists such as low-dose naltrexone may act centrally or peripherally to alter nociceptive and pruriceptive processing [14, 15, 18]. Although these mechanisms remain incompletely defined in SSc, the observed improvements in pruritus across select studies suggest that targeting immune and neuroimmune pathways may hold therapeutic relevance beyond effects on skin fibrosis alone.

Across the studied literature, some promising interventions were identified. Oral lenabasum demonstrated statistically significant improvement in pruritus as measured by the 5-D itch scale [14]. Although statistical significance was not demonstrated, low-dose naltrexone showed improvement in pruritus in a small case series of three patients using the 10-point FACES scale, suggesting a potential role for opioid modulation in SSc-related itch [18]. Additionally, rituximab demonstrated qualitative improvement in pruritus, although outcomes were reported descriptively without validated pruritus instruments [20]. In contrast, ketotifen did not demonstrate improvement in pruritus compared with placebo. Although ketotifen is commonly used to inhibit mast cell histamine release, this study did not demonstrate significant inhibition of mast cell degranulation at the evaluated dosage [23].

Taken together, the findings of this review highlight the clinical relevance of pruritus as a symptom that warrants greater attention in both research and clinical practice. Given the significant impact of chronic itch on quality of life, sleep, and psychological well-being, incorporation of pruritus as a prespecified outcome in systemic sclerosis trials may provide a more comprehensive assessment of treatment benefit from the patient perspective. Future studies should prioritize validated pruritus measures, explore mechanistic correlates of itch severity, and evaluate therapies designed specifically to address SSc-associated pruritus.

It is important to note that studies concerning localized scleroderma (LS) were also identified and analyzed during the initial literature. Several identified therapies showed improvement in pruritus-related outcomes in LS. These studies were excluded from the final analysis due to the clinically distinct pathophysiology of localized scleroderma from systemic sclerosis, lacking the systemic autoimmune and internal organ involvement characteristic of SSc.

This systematic review is limited by small sample sizes, reliance on case series, variability in pruritus scales, and heterogeneity in itch measurements. Several included studies assessed pruritus qualitatively or used heterogeneous measurement tools, limiting comparability across treatment regimens. Most studies also did not primarily assess pruritus, limiting the consistency of data and its interpretability. Future studies should include pruritus as a prespecified outcome in SSc trials using validated measurement tools (such as 5-D itch scale or VAS) and adequate sample size. Further investigation into neuroimmune mediators and signaling pathways may help identify targeted therapies for SSc-associated pruritus.

**Table 1:**
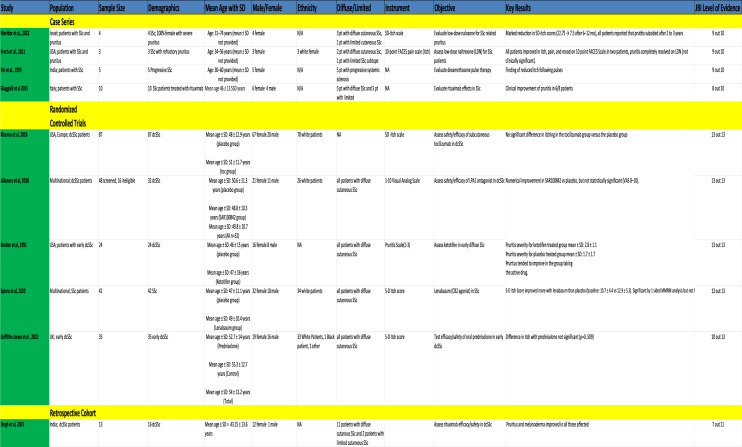
Studies used for systematic review
